# Intravenous Propofol Allows Fast Intubation in Neonates and Young Infants Undergoing Major Surgery

**DOI:** 10.3389/fped.2019.00321

**Published:** 2019-08-14

**Authors:** Stefania Sgrò, Francesco Morini, Patrizia Bozza, Fiammetta Piersigilli, Pietro Bagolan, Sergio Picardo

**Affiliations:** ^1^Department of Anesthesia and Critical Care, Ospedale Pediatrico Bambino Gesù, IRCCS, Rome, Italy; ^2^Department of Medical and Surgical Neonatology, Ospedale Pediatrico Bambino Gesù, IRCCS, Rome, Italy

**Keywords:** propofol, sevoflurane, neonates, induction agent, intubation

## Abstract

**Aim of the study:** In selected surgical neonates and infants, the rapidity of induction and intubation may represent an important factor for their safety. Propofol is an anesthetic characterized by a rapid onset and fast recovery time that may reduce time of anesthetic induction and improve post-anesthetic outcome. The aim of this study was to evaluate the safety and efficacy of anesthesia induction in full-term neonates and young infants after propofol bolus administration.

**Methods:** A retrospective case-control study including infants below 6 months of age, undergoing general anesthesia between 2011 and 2013, was carried out. Patients that received intravenous propofol bolus to induce anesthesia were compared to patients who received inhaled sevoflurane. Time to reach successful orotracheal intubation (OTI) was measured in seconds. The quality of OTI was defined as “excellent,” “good,” and “poor,” based on established classification and was reported. Hemodynamic parameters as systolic blood pressure (SBP), diastolic blood pressure (DBP), pulse pressure (PP), heart rate (HR), and oxygen saturation (SaO2) were collected before OTI (t0), at OTI (t1), and at spontaneous breathing recovery (t2). Main adverse effects were recorded for both groups. Results are median (IQ range) or prevalence; *p* < 0.05 was considered significant.

**Results:** 160 infants were enrolled in the study, 80 received propofol and 80 inhaled sevoflurane. Major surgery (involving organs in the thoracic, abdominal, or pelvic cavities) was performed in 64 and 54% of patients in the propofol and sevoflurane group, respectively (*p* = 0.07). Patients in the propofol group showed a shorter time for OTI [11.5 (4.0–65) vs. 360.0 (228.0–720.0) seconds, (*p* < 0.0001)]. No difference was found in the quality of OTI between the two groups. No significant complications were recorded in either group.

**Conclusions:** Propofol is a safe and effective anesthetic in neonates and infants permitting rapid induction of anesthesia and rapid intubation, without negative impact on the quality of intubation and haemodynamic compromise.

## Introduction

Neonates and infants are often intubated before undergoing surgery. Prolonged induction and intubation time may be associated with adverse effects such as hemodynamic instability and the development of hypoxemia, especially in infants, and neonates (1). Shorter duration of anesthesia induction and postoperative fast recovery time may be key determinants of improved outcomes particularly in neonates and infants.

Propofol is a soluble fat alkyl phenol with hypnotic properties commonly used to induce anesthesia in children and adults (2). Due to its pharmacokinetic properties propofol has been proposed as an agent with a very rapid onset and a fast recovery time, making it potentially well-suited for rapid induction of anesthesia, however little attention has been paid to its use in neonates and infants (3–5).

We hypothesized that iv propofol bolus may reduce anesthesia induction in neonates and infants undergoing elective major surgery.

## Materials and Methods

This is a retrospective case-control study conducted at the Bambino Gesù Children's Hospital, in Rome, Italy, based on an institutional protocol used as a standard of care to induce anesthesia. As propofol was already in clinical use, no approval from the Ethical Committee was required from our Institution for this study.

Neonates within 28 days of life and infants of <6 months of age undergoing elective surgery between January 2011 and April 2013 were included. Surgery included abdominal, pelvic, and thoracic major procedures. For every patient who underwent anesthesia induction with propofol, one patient who underwent induction with sevoflurane during the same period with comparable age was included in the study. An attempt was made to have also the same type of surgical disease in cases and controls, but this was not always possible.

Neonates and infants with significant structural congenital heart disease, intracranial malformation, preexisting hemodynamic instability, history of seizures, opioids, and/or hypnotic administration and prematurity (defined as gestational age <37 weeks) were excluded.

### Anesthesia Induction

Many anesthetic agents are off-label for neonates and infants. The use of one or more agents is often based on established clinical practice, experience, availability, and limited data. The American Society of Anesthesiologists has published practice guidelines for acute pain management in the perioperative setting suggesting that each center must establish its own set of standard protocols to optimize patient care (6). In our Hospital, cases were managed by an anesthesiologist team according to an institutional treatment. In brief, the protocol includes the use of propofol or sevoflurane as a standard of care based on the anesthesiologist clinical evaluation to anesthesia induction.

Anesthesia was performed by the same experienced anesthesiologist (SS) in all patients. In the propofol Group, all patients had a venous cannula *in situ*. Propofol (0.5%) was administered intravenously (iv) at 4 mg/kg over 15 s (7). In the control group, inhaled sevoflurane at 4% was administered. The dose of sevoflurane was the measured inspiratory concentration. No neuromuscular blocking agents were used in either groups.

Clinical requirement for additional boluses of propofol or sevoflurane administration were recorded. Time to reach successful orotracheal intubation (OTI) was measured in seconds and recorded routinely. For sevoflurane, the time to reach successful OTI was measured from the moment when the concentration in inspired gases was 4% until the moment when the tube was placed below the vocal cords. For propofol, the time to reach successful OTI was measured from the end of propofol infusion until the moment when the tube was placed below the vocal cords.

### Hemodynamic Parameters

Systolic blood pressure (SBP), diastolic blood pressure (DBP), pulse pressure (PP), heart rate (HR), and transcutaneous oxygen saturation (SpO2) were routinely measured non-invasively every 10 min and reported at t0 (before OTI), t1 (at OTI) and t2 (spontaneous breathing recovery). Hypotension was defined as a reduction of systolic blood pressure (SBP) > 25 mmHg compared to initial values. Hypotension was treated by a standard protocol of initial saline bolus at 10 ml/kg. Bradycardia was defined as HR < 100 bpm for at least 60 s and desaturation as SpO2 <85% for at least 60 s.

### Quality of OTI and Adverse Effects

Orotracheal intubation was performed by the same experienced anesthesiologist (SS) using #0 Macintosh laryngoscope blade. The quality of OTI was graded according to the Good Clinical Research Practice (GCRP) criteria (Table 1) (8). The evaluation of quality was classified as excellent if all the parameters were excellent, good if all the parameters were either excellent or good, and poor if only one parameter was described as poor. All adverse effects were reported, including potential side effects of seizures, skin rashes, and laryngeal spasm.

**Table 1:** Parameters and classification of the quality of intubation.

**Parameter analyzed**	**Excellent**	**Good**	**Poor**
Laringoscopy	Easy	Average	Difficult
Vocal chords position	Open	Intermediate	Closed
Vocal chords movements	Absent	In movement	Closed
Spontaneous limb movements	Absent	Slight	Vigorous
Cough	Absent	Diaphragmatic	sustained

### Statistical Analysis

Comparison between groups was performed using Mann-Whitney and Fisher's exact test as appropriate. Data were analyzed using GraphPad Prism 5.0 Macintosh version (GraphPad Software, San Diego California USA, www.graphpad.com). *P* value <0.05 was considered significant.

## Results

One hundred and sixty cases were scheduled for elective surgery and included in this study. Of these 80 received propofol and 80 sevoflurane. Demographics data for both groups are reported in Table 2.

**Table 2:** Baseline demographic characteristics of each group.

	**Propofol (80 patients)**	**Sevoflurane (80 patients)**	***p***
Male/Female	38/42	37/43	0.8229
Gestational age (weeks)	37.8+/−1.4	38.1+/−1.2	0.428
Birth weight (kg)	3.2+/−0.5	3.2+/−0.5	0.5456
Major surgery	64%	54%	0.0687
Thorax/Abdomen	33/47	24/55	0.0478

*Results are mean ± standard deviation or prevalence*.

In the propofol group, 30 (38%) cases were neonates (15 males, M, and 15 females, F), 50 (62%) cases were infants (25 M/25 F). In the sevoflurane group 45 (56%) were neonates (12 M/33 F), 35 (44%) were infants (10 M/ 25 F) (Table 2). No significant demographic differences were found between the propofol and sevoflurane groups.

The time for OTI was significantly reduced in the propofol group compared to the sevoflurane group [median (IQ range): 11.5 (4.0–65) vs. 360.0 (228.0–720.0) s, *p* < 0.0001].

The quality of OTI results are summarized in Table 3. No difference was found in the quality of OTI between the two groups. For the propofol group 68 cases (85%, of which 39 neonates and 29 infants) showed an excellent quality for OTI and 12 (15%) a good quality. No cases reported poor quality. For the sevoflurane group, quality for OTI turned out to be excellent in 64 cases (80%, 28 neonates and 36 infants) and good in 11 cases (14%, 6 neonates and 5 infants). Five cases (6%, 3 neonates and 2 infants) had poor OTI.

**Table 3:** Comparison of the quality of intubation between infants induced with propofol and sevoflurane.

	**Excellent**	**Good**	**Poor**	**Additional bolus**
Propofol (*n*)	68	12	0	7
(neonates/infants)	39/29	8/4		6/1
Sevoflurane (*n*)	64	11	5	0
(neonates/infants)	38/36	6/5	3/2	

In the propofol group, 7 cases (9%, 6 neonates and 1 infant) required an additional bolus of propofol to obtain adequate anesthesia. In the sevoflurane group none required additional doses.

Transient hypotension, spontaneously resolving with no need for pharmacologic therapy, was observed in two cases (both infants) in the propofol group and in one case in the sevoflurane group. No episodes of desaturation or bradycardia were observed in the two groups.

In the propofol group two patients developed tonic-clonic seizures (1 neonate and 1 infant) which resolved spontaneously after few seconds, and three developed a skin rash (2 neonates and 1 infant). In the sevoflurane group two cases of laryngeal spasm occurred.

## Discussion

In the present study we compared the use of iv propofol bolus to sevoflurane inhalation for anesthetic induction in neonates and infants undergoing major surgery. We found that propofol significantly reduced time for intubation compared to inhaled sevoflurane. Propofol use was not associated with an increased risk of adverse effects and showed similar hemodynamic effects and quality of OTI compared to sevoflurane.

A “modified” version of rapid sequence induction has been proposed in pediatric patients, in order to increase the safety of patients considered at risk of pulmonary aspiration of gastric contents and to reduce hemodynamic negative effects (9). Several pharmacologic agents have been used to facilitate rapid successful OTI and post-anesthetic recovery (10), but despite significant investigations, there is still no standard consensus and a lack of large randomized controlled trials in neonates and infants. Morimoto and coworkers, in a randomized controlled study (11), analyzed 8% Sevoflurane and 4% Halotane for rapid induction of anesthesia in children, concluding that sevoflurane can be safely used for rapid induction of anesthesia in children. However, the use of sevoflurane may be associated with reduced SBP, especially in younger children (12), as well as heart rhythm abnormalities such as tachycardia and QTc prolongation (11–13). Therefore, the need for further alternatives. Propofol is a lipid-soluble anesthetic agent with a very rapid onset and fast recovery time, making it potentially well-suited for anesthesia induction and successful intubation in neonates. In preterm infants undergoing intubation propofol has been associated with shorter procedure time and faster recovery time (14–16). Our results showed a shorter time to reach successful intubation. Similar findings were reported by Ghanta et al. (4) comparing propofol (2.5 mg/kg) to morphine, atropine, and suxamethonium in a cohort of 63 neonates. In the present study, higher doses of propofol were administered, as compared to older children (4 vs. 2 mg/kg). The need for higher propofol dose may be explained by an immature enzymatic system in neonates (17–21). Neonates are characterized by an immature enzymatic system resulting in different pharmacokinetic and pharmacodynamic properties, as well as altered distribution and clearance compared to older children (17–21). Propofol is predominantly metabolized in the liver, by the phase II enzyme UDP-glucoronosyltransferase. Supporting this hypothesis, Anderson et al. described a decreased ability for glucuronidation during the neonatal period, particularly in phase II reaction, suggesting that perhaps higher propofol dosage may be necessary to obtain adequate sedation. Another mechanism which may explain the need for higher doses is an increased total amount of water in neonates, affecting the distribution of drugs, propofol distribution, and concentration (22). Additionally, inter-individual variability in propofol metabolism may contribute to altered distribution and should be considered too (19).

Propofol use for intubation in newborn infants has also been associated with systemic hypotension particularly when used at higher doses (7, 16). The mechanisms of propofol-induced hypotension may be caused by a direct systemic vasodilation and a negative inotropic effect on the myocardium (23). The latter may be due to reduced intracellular Ca2+ availability, through the sarcoplasmatic reticulum, leading to myocardial impairment (24, 25). In our study, propofol bolus administration at 4 mg/kg had limited hemodynamic effects. Only three cases developed mild hypotension, which resolved spontaneously suggesting perhaps a transient effect. Mild hypotension, of limited clinical significance, has also been described among full term neonates and infants who received propofol (26–29). The haemodynamic effects of propofol remain incompletely understood and need further investigation.

This study has some limitations. We used retrospective data, and cases were not randomly assigned to each group but based on subjective clinical judgment. Another limitation is that per Institutional protocol we use 4% sevoflurane, while in children higher concentrations have been reported (up to 8%) (30), and this may fasten the induction phase. For the neonate, different protocols, with different sevoflurane concentrations, are reported (17, 31). However, no specific guidelines exist for neonatal anesthesia and most of the anesthetic agents used in clinical practice, including Sevoflurane, have not been tested thoroughly for their safety in neonates. Therefore, we followed the prudential concept of limiting the doses and exposure of single or multiple drugs in our patients.

In conclusion, as compared to sevoflurane, propofol allows quicker induction and intubation, without negative effects on the quality of OTI or the hemodynamic parameters. Our findings may be relevant for the future of rapid sequence induction and intubation in neonates and small infants, maintaining hemodynamic stability and quality of intubation. Propofol administration may represent a valid option compared to sevoflurane 4% to anesthesia induction. Neonates, infants, and small children have, compared with adult patients, a reduced tolerance to apnea because of limited cooperation during preoxygenation, reduced functional residual capacity and increased oxygen demand. The use of a faster drug may have a positive impact, accelerating the intubation time. Despite significant hemodynamic complications which did not occur in our series, we suggest that in neonates and small infants propofol is used by experienced anesthesiologists and under strict hemodynamic monitoring. Further prospective studies, and larger samples, may help to confirm the safety and efficacy of propofol in this population, and to define if higher Sevoflurane concentrations compare better to propofol in terms of safety and efficacy in neonatal anesthesia induction.

## Ethics Statement

The study was on two different standard practices: no Ethical Committee approval needed. This is a retrospective study and no specific informed consent was required from the patients caregivers for the study. Patients caregivers gave written informed consent to anesthetic drugs used in present study, and retrospectively analyzed.

## Author Contributions

SS conceptualized the study, collected patients' data, drafted the manuscript and approved the final version of the manucript. FM and FP analyzed the data, revised the manuscript and approved the final version of the manuscript. PaB and PiB revised the manuscript and approved the final version of the manuscript. SP participated in the concept of the study, revised the manuscript and approved the final version of the manuscript.

### Conflict of Interest Statement

The authors declare that the research was conducted in the absence of any commercial or financial relationships that could be construed as a potential conflict of interest. The reviewer PM declared a past co-authorship with one of the authors PiB to the handling editor.
